# NF-*κ*B mediates the 12(S)-HETE-induced endothelial to mesenchymal transition of lymphendothelial cells during the intravasation of breast carcinoma cells

**DOI:** 10.1038/bjc.2011.194

**Published:** 2011-05-31

**Authors:** C Vonach, K Viola, B Giessrigl, N Huttary, I Raab, R Kalt, S Krieger, T P N Vo, S Madlener, S Bauer, B Marian, M Hämmerle, N Kretschy, M Teichmann, B Hantusch, S Stary, C Unger, M Seelinger, A Eger, R Mader, W Jäger, W Schmidt, M Grusch, H Dolznig, W Mikulits, G Krupitza

**Affiliations:** 1 Institute of Clinical Pathology, Medical University of Vienna, Waehringer Guertel 18-20, A-1090 Vienna, Austria; 2 Department of Medicine I, Institute of Cancer Research, Medical University of Vienna, Vienna, Austria; 3 University of Applied Science, Krems, Austria; 4 Department of Clinical Pharmacy and Diagnostics, University of Vienna, Vienna, Austria; 5 Neuromuscular Research Department, Center for Anatomy and Cell Biology, Medical University of Vienna, Vienna, Austria; 6 Institute of Medical Genetics, Medical University of Vienna, Vienna, Austria

**Keywords:** LEC motility, VE-cadherin, ZEB1, S100A, NF-*κ*B

## Abstract

**Background:**

The intravasation of breast cancer into the lymphendothelium is an early step of metastasis. Little is known about the mechanisms of bulky cancer invasion into lymph ducts.

**Methods:**

To particularly address this issue, we developed a 3-dimensional co-culture model involving MCF-7 breast cancer cell spheroids and telomerase-immortalised human lymphendothelial cell (LEC) monolayers, which resembles intravasation *in vivo* and correlated the malignant phenotype with specific protein expression of LECs.

**Results:**

We show that tumour spheroids generate ‘circular chemorepellent-induced defects’ (CCID) in LEC monolayers through retraction of LECs, which was induced by 12(S)-hydroxyeicosatetraenoic acid (HETE) secreted by MCF-7 spheroids. This 12(S)-HETE-regulated retraction of LECs during intravasation particularly allowed us to investigate the key regulators involved in the motility and plasticity of LECs. In all, 12(S)-HETE induced pro-metastatic protein expression patterns and showed NF-*κ*B-dependent up-regulation of the mesenchymal marker protein S100A4 and of transcriptional repressor ZEB1 concomittant with down-regulation of the endothelial adherence junction component VE-cadherin. This was in accordance with ∼50% attenuation of CCID formation by treatment of cells with 10 *μ*M Bay11-7082. Notably, 12(S)-HETE-induced VE-cadherin repression was regulated by either NF-*κ*B or by ZEB1 since ZEB1 siRNA knockdown abrogated not only 12(S)-HETE-mediated VE-cadherin repression but inhibited VE-cadherin expression in general.

**Interpretation:**

These data suggest an endothelial to mesenchymal transition-like process of LECs, which induces single cell motility during endothelial transmigration of breast carcinoma cells. In conclusion, this study demonstrates that the 12(S)-HETE-induced intravasation of MCF-7 spheroids through LECs require an NF-*κ*B-dependent process of LECs triggering the disintegration of cell–cell contacts, migration, and the generation of CCID.

Breast cancer is the most common malignancy causing the highest death rate among women. Noteworthy, patients are not threatened by the primary tumour, but by metastases that destroy the function of infested organs. Breast cancer is believed to spread mainly through the lymphatic vasculature and as soon as carcinoma cell emboli are detectable in intrametastatic lymphatic vessels of sentinel lymph nodes (intrametastatic carcinosis), the postsentinel lymph nodes also fill up with cancer cells (Kerjaschki *et al*, 2011). The number of metastasised lymph nodes is a clinical predictor for the development of distant organ metastases and patient outcome (Carlson *et al*, 2009). Hence, understanding early steps of tumour cell intravasation into the lymphatic vasculature is important for the development of tailored anti-metastatic treatment concepts. Ductal breast cancer accesses the lymphatics in bulks generating gaps in the lymphendothelial cell (LEC) wall that serve as entry gates for the tumour. Therefore, we aimed to investigate the mechanisms of breast cancer cells that generate gaps – and as we now call them – ‘circular chemorepellent-induced defects’ (CCID) into LEC monolayers to identify potential target molecules for therapy. In a 3-dimensional (3D) co-culture model *in vitro*, we recently demonstrated that human MCF-7 breast cancer spheroids induced the formation of CCID into LEC monolayers right underneath the spheroids through centrifugal LEC migration (Madlener *et al*, 2010), a process closely resembling the situation in human patients. Tumour cells (MCF-7) secrete 12(S)-hydroxyeicosatetraenoic acid (HETE) (Uchide *et al*, 2007), which is produced by lipoxygenase-15 (ALOX15) in MCF-7 cells. Our recent study identified this arachidonic acid metabolite as one of the major factors in the process of CCID formation (Kerjaschki *et al*, 2011). Notably, 12(S)-HETE was described as the ‘endothelial retraction factor’ (Honn *et al*, 1994). The NF-*κ*B promotes endothelial cell migration (Flister *et al*, 2010) and in preliminary experiments, we found that NF-*κ*B inhibition reduced CCID formation. As the migration of LECs is an early and relevant event in mammary tumour cell intravasation and metastasis, we investigated the mechanism of 12(S)-HETE and the role of NF-*κ*B on LEC motility.

## Materials and methods

### Chemicals

The I-*κ*B*α* phosphorylation inhibitor (E)-3-[(4-methylphenylsulfonyl]-2-propenenitrile (Bay11-7082) was from Biomol (Hamburg, Germany) and 12(S)-HETE was purchased from Cayman Chemical (Ann Arbor, MI, USA).

Monoclonal antibody against CD144 (VE-cadherin) (PN IM1597) was from Beckman Coulter (Fullerton, CA, USA). The polyclonal rabbit anti-paxillin antibody (H-114) (SC-5574), the monoclonal mouse *α*-tubulin (DM1A) antibody, and rabbit polyclonal anti-ZEB1 (H-102) were purchased from Santa Cruz Biotechnology (Santa Cruz, CA, USA).

Monoclonal mouse antibody phospho-p44/42 MAPK (Erk1/2) (Thr202/Tyr204) (E10), monoclonal rabbit p44/42 MAPK (Erk1/2) (137F5) antibody, polyclonal rabbit antibody phospho-myosin light chain 2 (MLC2) (Ser19), polyclonal rabbit MLC2 antibody, monoclonal mouse antibody phospho-Akt (Ser473) (587F11), polyclonal rabbit Akt antibody, monoclonal rabbit antibody ROCK-1 (C8F7), polyclonal rabbit ILK1 antibody, and polyclonal rabbit MYPT1 antibody were from Cell Signaling (Danvers, MA, USA). Monoclonal mouse anti-*β*-actin (clone AC-15) and monoclonal mouse anti-acetylated-tubulin (clone 6-11B-1) were from Sigma-Aldrich (Munich, Germany). The polyclonal rabbit IgG anti-phospho-MYPT1 (Thr696) was purchased from Upstate (Lake Placid, NY, USA). The polyclonal rabbit phospho-specific actin (Tyr-53) antibody was from extracellular matrix (ECM) Biosciences (Versailles, KY, USA). Rabbit anti-S100A4 was purchased from Sigma (St Louis, MO, USA). Polyclonal goat ARP2/3 subunit 1B antibody was purchased from Abcam (Cambridge, MA, USA). Polyclonal rabbit anti-mouse and anti-rabbit IgGs were from Dako (Glostrup, Denmark). Alexa-Fluor 488 (green) goat-anti-rabbit and Alexa-Fluor 594 (red) goat-anti-mouse labelled antibodies were purchased from Molecular Probes, Invitrogen (Karlsruhe, Germany).

### Cell culture

Human MCF-7 breast cancer cells were grown in MEM medium supplemented with 10% fetal calf serum (FCS), 1% penicillin/streptomycin, 1% NEAA (Invitrogen) at 37°C in a humidified atmosphere containing 5% CO_2_. Telomerase-immortalised human LECs were grown in EGM2 MV (Clonetics, Allendale, NJ, USA) at 37°C in a humidified atmosphere containing 5% CO_2_.

For gap formation assays, LECs were stained with cytotracker green purchased from Invitrogen.

### 3D co-cultivation of MCF-7 cancer cells with LECs

Mock cells (MCF-7) were transferred to 30 ml MEM medium containing 6 ml of a 1.6% methylcellulose solution (0.3% final concentration; Cat. No.: M-512, 4000 centipoises; Sigma, Karlsruhe, Germany). A total of 150 *μ*l of this cell suspension were transferred to each well of a 96-well plate (Greiner Bio-one, Cellstar 650185, Kremsmünster, Austria) to allow spheroid formation within the following 2 days. Then, MCF-7 spheroids were washed in phosphate-buffered saline (PBS) and transferred to cytotracker-stained LEC monolayers that were seeded into 24-well plates (Costar 3524, Sigma-Aldrich) in 2 ml EGM2 MV medium.

### CCID assay

The MCF-7 cell spheroids (3000 cells/spheroid) were transferred to the 24-well plate containing LEC monolayers. After 4 h of incubating the MCF-7 spheroids-LEC monolayer co-cultures, the gap sizes in the LEC monolayer underneath the MCF-7 spheroids were photographed using an Axiovert (Zeiss, Jena, Germany) fluorescence microscope to visualise cytotracker(green)-stained LECs underneath the spheroids. Gap areas were calculated with the Axiovision Re. 4.5 software (Carl Zeiss, Jena, Germany). The MCF-7 spheroids were treated with solvent (DMSO) as negative control. Each experiment was performed in triplicate and for each condition, the gap size of 12 and more spheroids was measured.

### Confocal microscopy and immunofluoresce analysis

Lab-Tek II chambered coverglasses (Nalgen Nunc International, Wiesbaden, Germany) were coated with 10 *μ*g ml^–1^ fibronectin for 1 h at room temperature. Lymphendothelial cells were seeded in 1 ml EGM 2 MV onto chambered coverslips and allowed to grow for 2 days followed by co-cultivation with MCF-7 spheroids on LEC monolayers. After 4 h of incubation, cells were washed with ice-cold PBS and fixed in 4% paraformaldehyde for 15 min at room temperature. Cells were immunostained with various antibodies and analysed by confocal microscopy. For this, cells were washed with PBS and permeabilised with 0.1% Triton X-100 in PBS for 30 min at room temperature, followed by washing with PBS and blocking for 1 h with 10% goat serum diluted in BSA. Thereafter, the cells were incubated with the primary antibody against VE-cadherin diluted 1 : 50 for 1 h at room temperature and washed with PBS. Cells were further incubated with a fluorescence labelled second antibody diluted 1 : 1000 for 1 h at room temperature in the dark and washed with PBS. Cells were counterstained with DAPI (dilution 1 : 50 0000) at room temperature.

### Western blotting

Lymphendothelial cells were seeded in 6 cm dishes and treated with the indicated compounds (10 *μ*M Bay11-7082 and or 1 *μ*M 12(S)-HETE). Cells were washed twice with ice-cold PBS and lysed in buffer containing 150 mM NaCl, 50 mM Tris pH 8.0, 0,1% Triton X-100, 1 mM phenylmethylsulfonylfluorid and protease inhibitor cocktail. Afterwards, the lysate was centrifuged at 12 000 r.p.m. for 20 min at 4°C and the supernatant was stored at −20°C until further analysis. Equal amounts of protein samples were separated by SDS polyacrylamide gel electrophoresis and electro-transferred onto Hybond PVDF membranes at 100 V for 1 h at 4°C. To control equal sample loading, membranes were stained with Ponceau S. After washing with PBS/T (PBS/Tween 20; pH: 7.2) or TBS/T (Tris-buffered saline/Tween 20; pH: 7.6), membranes were immersed in blocking solution (5% non-fat dry milk in TBS containing 0.1% Tween or in PBS containing 0.5% Tween 20) at room temperature for 1 h. Membranes were washed and incubated with the first antibody (in blocking solution; dilution 1 : 500–1 : 1000) by gently rocking at 4°C overnight or at room temperature for 1 h. Thereafter, the membranes were washed with PBS/T or TBS/T and incubated with the second antibody (peroxidase-conjugated goat-anti-rabbit IgG or anti-mouse IgG; dilution 1 : 2000) at room temperature for 1 h. Chemiluminescence was detected by ECL detection kit (Thermo Scientific, Portsmouth, NH, USA) and the membranes were exposed to Amersham Hyperfilms (GE-Healthcare, Amersham, Buckinghamshire, UK).

### Transient siRNA transfection

Lymphendothelial cells were grown in 6-well plates to 70% confluence in EGM 2 MV medium. Cells were subsequently transfected using RNAiFect (Qiagen, Hamburg, Germany). siRNA (ZEB1 silencer select pre-designed siRNA ID: s13883, and ID: s13885, and scrambled RNA Ambion; Applied Biosystems, Austin, TX, USA) was diluted in culture medium containing FCS and antibiotics (final volume 100 *μ*l) to a final concentration of 100 nM. A total of 15 *μ*l of RNAiFect transfection reagent was added to the diluted siRNA and incubated for 15 min at room temperature. Then the mixture was added to cells and incubated for 8 h at 37°C. Thereafter, the medium was changed and the cells were incubated further 48 h. ZEB1 expression was analysed by western blotting.

### Statistical analysis

Dose–response curves were analysed using Prism 4 software (San Diego, CA, USA) and significance was determined by paired Student's *t*-test. Significant differences between experimental groups were ^*^P<0.05.

## Results

### 12(S)-HETE induces protein expression in LECs associated with motility

**Figure 1:**
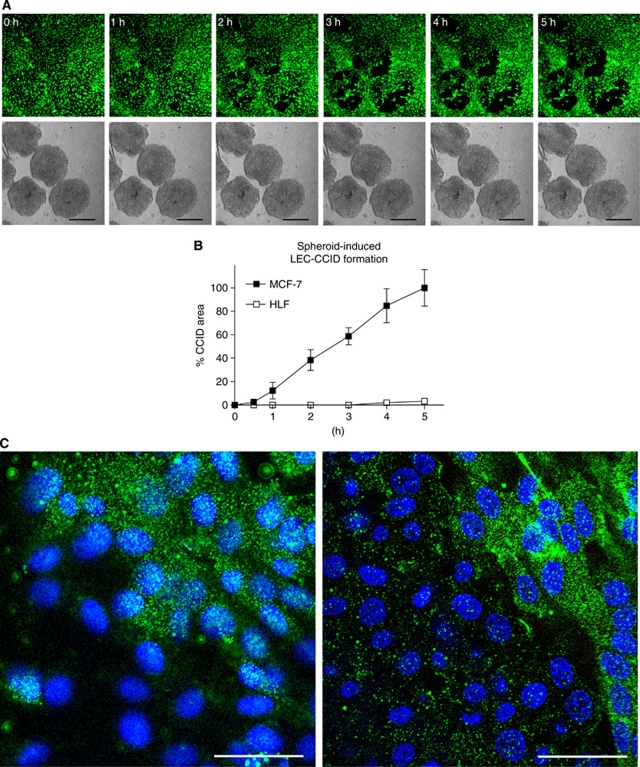
CCID formation by cell migration. (**A**) Time lap experiment show the same microscopic power field after 0–5 h co-culture of LECs (upper panel; cytotracker green, FITC filter) and MCF-7 spheroids (lower panel; phase contrast); The images show the progression of CCID formation over time. No apoptotic features were observed. Scale bars: 200 *μ*m. (**B**) The gradual increase of CCID areas over time was measured underneath five MCF-7 spheroids or human normal lung fibroblast spheroids (HLF) after the indicated time points using Axiovision software (Zeiss). Error bars indicate s.e.m. (**C**) LECs were grown on coverslips until confluence when MCF-7 spheroids were transferred on top of LECs and co-incubated for 4 h at 37°C to allow CCID formation. LECs were stained with respective antibodies. Confocal laser scanning microscopy of immunocytochemically stained LECs at the rim of CCID (upper right diagon each, which was the part covered by the MCF-7 spheroid) show elevated levels of phosphorylation (green; FITC filter) of MYPT threonine-696 (left panel) and MLC2 serine-19 (right panel), indicating increased cell mobility. Nuclei are stained with DAPI (blue). Scale bars: 45 *μ*m.

**Figure 2:**
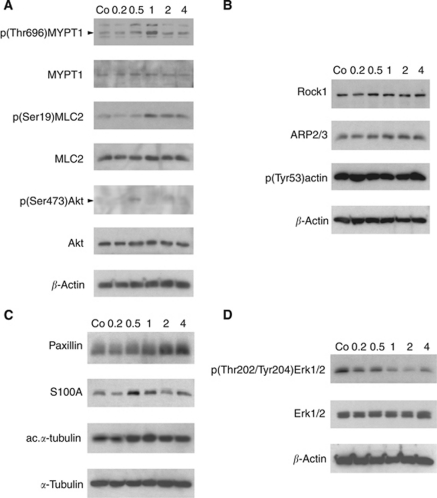
Modulation of protein expression and posttranslational modifications in LECs. LEC monolayers were incubated with 1 *μ*M synthetic 12(S)-HETE and analysed by western blotting after 0.2, 0.5, 1.0, 2.0, and 4.0 h. Equal sample loading was controlled by Ponceau S staining, *β*-actin (**A**, **B**, **D**), or *α*-tubulin (**C**) expression. Co, untreated LECs.

Breast cancer cells (MCF-7) secrete 12(S)-HETE (Uchide *et al*, 2007), which has been shown to induce the motility of endothelial cells (Honn *et al*, 1994). The time-dependent formation of CCIDs was caused by MCF-7 spheroids in the underneath growing LEC monolayer (Figures 1A and B). We could demonstrate by time lap microscopy that MCF-7 spheroid-induced CCID formation was the result of rapid cell retraction rather than a cell clearence through apoptosis (Kerjaschki *et al*, 2011). Confocal laser scanning microscopy revealed that cell retraction correlated with the increased phosphorylation of myosin light chain phospho-transferase (MYPT1, synonym: PPP1R12A) threonine-696 and of MLC2 serine-19 in underneath growing LECs at the rim of CCIDs (Figure 1C; upper right corner each, which was covered by the MCF-7 spheroid), indicating a mobile LEC phenotype. To simplify the 3D co-culture model consisting of MCF-7 spheroids and LEC monolayer, in which the role of ALOX15, ALOX12, and 12(S)-HETE was investigated in detail (Madlener *et al*, 2010; Kerjaschki *et al*, 2011) and to analyse protein expression/activation, LECs were treated with 1 *μ*M synthetic 12(S)-HETE. Indeed, purified 12(S)-HETE increased the phosphorylation of MYPT1 in LECs within 1 h (Figure 2A), confirming our recent data (Kerjaschki *et al*, 2011). Furthermore, MLC2 showed increased phosphorylation, which substantiated the fact that 12(S)-HETE induced the motility of LECs.

Akt is an important component in pro-survival pathways but also significantly involved in pro-migratory signalling (Burgering and Coffer, 1995; Franke *et al*, 1997). Treatment with 12(S)-HETE transiently increased the level of phosphorylated Akt within 30 min (Figure 2A).

Arp2/3 activity correlates with mesenchymal-type migration, whereas ROCK-1 is associated with amoeboid migration (Paulitschke *et al*, 2010) and both co-regulate the actin cytoskeleton (Xu *et al*, 2009; To *et al*, 2010). 12(S)-HETE stimulated a marginal increase of ROCK-1 and Arp2/3 expression; however, the constitutive phosphorylation of actin at the Tyr53 activation site remained unchanged (Figure 2B).

Paxillin is a focal adhesion phosphoprotein contributing to the contact between the endothelial cell and the ECM, and its up-regulation associates with a mobile cell phenotype (Huang *et al*, 2003; Webb *et al*, 2004). Treatment of LECs with 12(S)-HETE caused an increase of paxillin after 2 h (Figure 2C) and a transient up-regulation of the pro-metastatic Ca^2+^ signal transducer S100A4, both suggesting a mesenchymal and mobile phenotype (Zeisberg and Neilson, 2009). S100A4 expression was reported to correlate with tubulin polymerisation (Lakshmi *et al*, 1993), which is indicated by increased acetylation of *α*-tubulin (Piperno and Fuller, 1985). In all, 12(S)-HETE slightly increased tubulin acetylation (Figure 2C) concomittant with S100A4 up-regulation and this was accompanied by dephosphorylation (inactivation) of Erk1/2 (Figure 2D). Active Erk and paxillin mediate disadhesion, a process required for a directionally migrating cell phenotype (Webb *et al*, 2004). The reason for 12(S)-HETE-mediated Erk inactivation upon treatment remains obscure. It might indicate that the migratory stimulus was not an attracting one, but a repelling one, or that 12(S)-HETE-induced LEC adhesion disassemby is independent of Erk. Yet, from the total of the data we conclude that 12(S)-HETE induced a mesenchymal and mobile LEC phenotype mandatory for metastatic intravasation.

### 12(S)-HETE transiently inhibits VE-cadherin expression and induces endothelial disassembly

**Figure 3:**
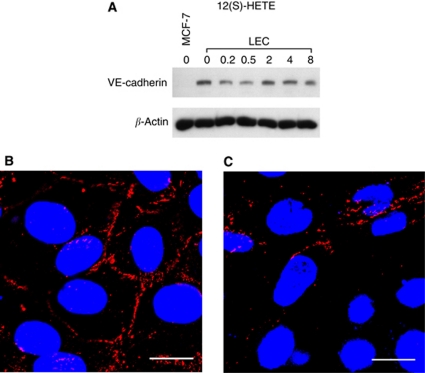
Analysis of VE-cadherin expression in LECs. (**A**) LECs were treated with 1 *μ*M 12(S)-HETE for 0.2, 0.5, 2, 4, and 8 h. Then, cells were harvested and protein lysates were analysed by western blotting. MCF-7 cells were used as negative control. Equal sample loading was controlled by Ponceau S staining and *β*-actin analysis. Confocal immunofluorescence images of LECs next to a spheroid (**B**) and underneath an MCF-7 spheroid (**C**). LECs were grown on coverslips until confluence when MCF-7 spheroids were transferred on top of LECs and co-incubated for 4 h at 37°C to allow CCID formation. LECs were stained with anti-VE-cadherin antibody (red) and DAPI (blue). (**B**) Distant to a spheroid, VE-cadherin structures appear well developed, whereas (**C**) VE-cadherin interactions are disrupted underneath an MCF-7 spheroid. Scale bar: 15 *μ*M. The colour reproduction of this figure is available at the *British Journal of Cancer* journal online.

For cell–cell cohesion, VE-cadherin is necessary and hence, for vascular integrity. Therefore, VE-cadherin is a marker for an endothelial, immobile phenotype that withstands metastatic cell intravasation. Conversely, metastatic cells have to interefere with VE-cadherin function to facilitate the migration of LECs. In fact, treatment of LECs with 12(S)-HETE transiently down-regulated VE-cadherin expression (Figure 3A).

To investigate the effect of MCF-7 spheroids on VE-cadherin expression of underneath LECs, we analysed VE-cadherin distribution by confocal immunofluorescence microscopy. Lymphendothelial cells at distance of MCF-7 spheroids showed intact VE-cadherin structures (Figure 3B). At the margin of CCID, LECs showed disintegrated and reduced VE-cadherin at cell boundaries, suggesting disassembly of endothelial organisation (Figure 3C). The MCF-7 cells constantly produce 12(S)-HETE and, therefore, the down-regulation of VE-cadherin of underneath growing LECs was observed even after 4 h of co-culture and was not only transiently suppressed as seen upon synthetic 12(S)-HETE treatment.

These data implicate that LEC motility might be caused by the loss of cell–cell contacts through down-regulation of VE-cadherin and suggest an endothelial to mesenchymal transition (EMT)-like process, both by the spheroid as well as by 12(S)-HETE.

### ZEB1 contributes to 12(S)-HETE-induced VE-cadherin repression

**Figure 4:**
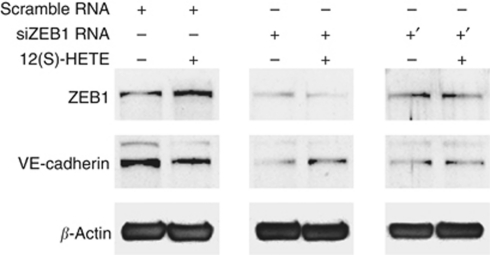
Effect of ZEB1 suppression on VE-cadherin regulation by 12(S)-HETE. LECs were transiently transfected with two different siRNAs against ZEB1 (+: siRNA1; +′: siRNA2), or with scrambled siRNA. LECs were subsequently treated with 1 *μ*M 12(S)-HETE and analysed by western blotting using antibodies against ZEB1 and VE-cadherin. Equal sample loading was controlled by *β*-actin expression.

E-cadherin is negatively regulated by the transcription factor and proto-oncogene ZEB1 (Eger *et al*, 2005; Chua *et al*, 2007; Peinado *et al*, 2007). Therefore, we examined whether VE-cadherin was also regulated by ZEB1. In fact, 12(S)-HETE rapidly induced ZEB1 that was accompanied by VE-cadherin repression (Figure 4). Since it was so far unknown whether ZEB1 also (co)regulates VE-cadherin, we investigated by siRNA approach whether knockdown of ZEB1 causes loss of VE-cadherin regulation by 12(S)-HETE. Two different and validated siRNAs were transiently transfected into LECs to specifically knockdown the expression of ZEB1. This resulted in the loss of VE-cadherin regulation upon 12(S)-HETE stimulation (Figure 4). Unexpectedly, blocking ZEB1 expression down-regulated constitutive VE-cadherin expression, which implicated that VE-cadherin was not directly regulated by ZEB1.

### Inhibition of NF-*κ*B blocks MCF-7-induced gap formation of LEC

**Figure 5:**
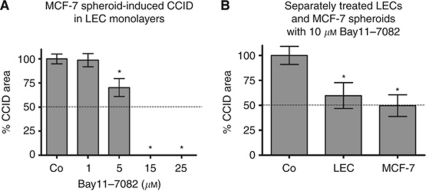
Quantitative analysis of formation and inhibition of CCID in LEC monolayers by MCF-7 spheroids formation. LECs were seeded into 24-well plates and allowed to grow for 2 days until confluence when LECs were stained with cytotracker green. (**A**) MCF-7spheroids, which were treated with different concentrations (solvent, 1, 5, 10, 15, and 25 *μ*M) of Bay11-7082 for 0.5 h at 37°C, were transferred on top of LECs. (**B**) Either LECs or MCF-7 spheroids were treated with the Bay11-7082 for 0.5 h, which was entirely washed off before both cell types were co-cultivated. The 3D-MCF-7 spheroids/LEC monolayer co-cultures were incubated for 4 h at 37°C. The size of CCIDs, which were formed by MCF-7 spheroids in the LEC monolayer in this time period, was measured using a Zeiss Axiovert microscope and Axiovision software. In the solvent treated controls, the CCID sizes in LEC monolayers were set 100%. For each condition, the gap area of at least 12 spheroids was measured. Error bars indicate standard error of the mean. Asterisks show significant differences in the inhibition of CCID formation compared with control (^*^P<0.05).

**Figure 6:**
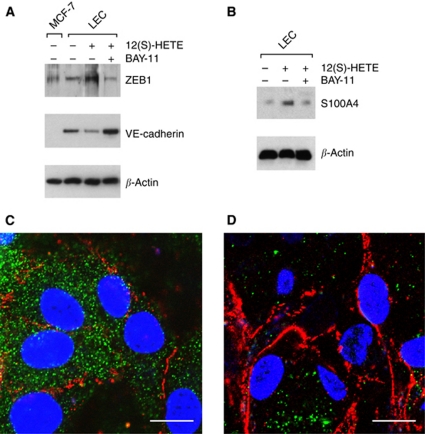
Analysis of mesenchymal marker expression in LECs after intervention with NF-*κ*B signalling. LECs were pretreated with 10 *μ*M of the I-*κ*B*α* phosphorylation inhibitor Bay11-7082 for 0.5 h and then stimulated with 1 *μ*M 12(S)-HETE for 0.2 h. Cells were harvested and analysed by western blotting using (**A**) anti-ZEB1 and anti-VE-cadherin antibodies. MCF-7 cells were used as negative control. (**B**) Blots were analysed with anti-S100A4 antibody. Equal sample loading was controlled by Ponceau S staining and *β*-actin analysis. Confocal immunofluorescence images of LECs at the rim of CCID (**C**) induced by an MCF-7 spheroid; (**D**) and from a similar position after treatment with 10 *μ*M Bay11-7082. LECs were grown on coverslips until confluence when MCF-7 spheroids were transferred on top of LECs and co-incubated for 4 h at 37°C to allow CCID formation. LECs were stained with anti-S400A4 antibody (green), anti-VE-cadherin antibody (red), and DAPI (blue). (**C**) S100A4 is well expressed and VE-cadherin interactions are disrupted. (**D**) Upon Bay11-7082 treatment, VE-cadherin structures again appear well developed (although unconnected to VE-cadherin structures of neighbouring cells), whereas S100A4 expression is decreased. Scale bar: 15 *μ*M.

The inhibition of NF-*κ*B translocation with Bay11-7082 blocked MCF-7 spheroid-induced gap formation of LECs in a dose-dependent fashion. A total of 10 *μ*M Bay11-7082 reduced CCID areas by 50–60% and 15 *μ*M prevented CCID formation almost completely (Figure 5A). Bay11-7082 is an irreversible inhibitor of I-*κ*B*α* phosphorylation and this allowed a specific experimental design that facilitated to discriminate whether NF-*κ*B activity of MCF-7 cells or of LECs contributed to CCID formation of LECs. Therefore, LEC monolayers or MCF-7 spheroids were each pretreated with Bay11-7082 for 30 min followed by a thorough washing procedure to prevent contaminating spill overs to the respective other cell type. Subsequently, MCF-7 spheroids were placed on the LEC monolayer (Figure 5B). Similar levels of inhibition were achieved when the drug was applied either on MCF-7 spheroids or on LECs, indicating that NF-*κ*B contributed to gap formation by at least two mechanisms. Here, we focussed only on the role of NF-*κ*B in LECs, regulating the change of endothelial plasticity associated with motility, and studied the expression of VE-cadherin and S100A4 by Western blot analysis. For this, LECs were pretreated with Bay11-7082 and then exposed to 12(S)-HETE. Bay11-7082 caused the up-regulation of VE-cadherin and the down-regulation of ZEB1 as well as of the mesenchymal marker protein S100A4 (Figures 6A and B). Immunocytochemistry confirmed that LECs expressed high levels of the mobility marker S100A4 (green) underneath MCF-7 spheroids (Figure 6C), which were down-regulated in the presence of Bay11-7082 (Figure 6D). Bay11-7082 prevented the suppression of VE-cadherin (red) underneath spheroids, although the VE-cadherin patterns appeared disintegrated and unconnected to adjacent cell borders (nuclei are in blue). These data suggest the involvement of NF-*κ*B in the acquisition of a mesenchymal-like phenotype of LECs, which induces single cell motility necessary for intravasation of breast carcinoma cells into the endothelium.

## Discussion

The progression of tumours to metastatic outgrowth is the fatal process of most cancer entities. Metastasis includes multiple steps such as intravasation of bulky tumours or dissociated single cells into the vasculature, transport through vessels, extravasation, invasion of tumour cells in target tissues, and manifestation of secondary tumours (Geiger and Peeper, 2009). Therefore, the direct interaction of tumour cells with vascular endothelial cells (Kramer and Nicolson, 1979) is one of the earliest events that facilitates intra- and extravasation into and from the blood or lymphatic vasculature (Honn *et al*, 1987). The break through of tumour emboli into intrametastatic lymphatic vessels of sentinel lymph nodes (Hirakawa *et al*, 2009) is the preceding step for the subsequent colonisation of lymph nodes along efferent axes with carcinoma cells. Notably, this event is indicative for a bad prognosis of ductal breast cancer (Kerjaschki *et al*, 2011). Hence, it is important to understand the mechanisms of tumour/lymphendothelial interactions. Here, we used a 3D-co-culture system to mimic an early step of trespassing breast cancer cells through the lymphatic vasculature. The generation of CCID into LEC monolayers recapitulated the situation in the sentinel and postsentinel lymph nodes in ductal breast cancer lymph metastasis in humans. Metastasis was shown to depend on the expression and activity of ALOXs that produce 12(S)-HETE as in case of MCF-7 spheroids (Uchide *et al*, 2007; Kerjaschki *et al*, 2011). In all, 12(S)-HETE induces endothelial cell retraction (Honn *et al*, 1994) and stimulates tumour cell spreading on the ECM (Timar *et al*, 1992). Several studies have shown the involvement of ALOXs in tumour differentiation and progression (Chen *et al*, 1994; Jiang *et al*, 2006; Nithipatikom *et al*, 2006) and increased levels of ALOX12 were observed in breast cancer (Jiang *et al*, 2006). We identified that LEC migration was the crucial step for CCID (Kerjaschki *et al*, 2011) and, therefore, LECs were treated with the pro-migratory factor 12(S)-HETE to analyse protein expression that causes or correlates with a mobile cell phenotype. Since 12(S)-HETE is a labile compound that is rapidly metabolised/degraded, the effects observed on protein expression were immediate (0.2–0.5 h) and transient. This was in contrast to the effects on LECs underneath spheroids, which were long lasting (4 h) due to the permanent supply of 12(S)-HETE by MCF-7 cells as *de novo* generated molecules.

Here, we demonstrated that MYPT1 and MLC2 became phosphorylated at the rim of MCF-7 spheroid-induced CCID in LECs. MYPT1 is the regulatory/targeting subunit of the myosin phosphatase, which regulates the interaction of actin and myosin in response to signalling through the GTPase Rho (Feng *et al*, 1999). Phosphorylation leads to the inhibition of MYPT1, cytoskeletal reorganisation and is associated with motility (Birukova *et al*, 2004a, 2004b). In addition, the phosphorylation of MLC2 at Thr18 and Ser19 (Ikebe and Hartshorne, 1985), which is correlated with myosin ATPase activity and contraction of myosine microfilament bundles (Tan *et al*, 1992), became induced in LECs upon 12(S)-HETE treatment, and also ROCK-1 became slightly up-regulated. ROCK is known to phosphorylate MLC2 at Ser19 regulating the assembly of stress fibres (Totsukawa *et al*, 2000) and causes focal adhesions generating an amoeboid movement (Sahai and Marshall, 2003). Moreover, Arp2/3, which levels were also marginally elevated by 12(S)-HETE, regulates mesenchymal invasion (Paulitschke *et al*, 2010).

The mobile state of induced LECs was furthermore confirmed by the increased expression of paxillin and protein S100A4. Paxillin (focal adhesion phosphoprotein) is necessary for cell–ECM contact, and its increased expression could already be associated *in vivo* and *in vitro* with enhanced endothelial cell motility (Lu *et al*, 2006; Deakin and Turner, 2008). S100A4 is a calcium-binding protein that interacts with intracellular target proteins (Mandinova *et al*, 1998) and is a marker for a mesenchymal phenotype and mesenchymal transition of epithelial cells, which encompasses cell mobility (Zeisberg and Neilson, 2009). In epithelial tumours, activation of the embryonic epithelial–mesenchymal transition programme is important for the dissemination and invasion of cancer cells (Yilmaz *et al*, 2007). S100A4 has been associated with migratory and invasive properties and is able to induce metastasis in rodent models of breast cancer (Rudland *et al*, 2000). Noteworthy, the levels of S100A4 mRNA are higher in breast carcinomas than in benign breast tumour specimens (Wang *et al*, 2000). S100A4 acts as an angiogenic factor by stimulating the motility and invasiveness of endothelial cells (Takenaga *et al*, 1994; Ambartsumian *et al*, 2001; Jenkinson *et al*, 2004; Schmidt-Hansen *et al*, 2004). Therefore, S100A4 has a role in both – cancer cells and endothelial cells – to increase malignancy.

Single cell motility can only be realised when cell–cell contacts of the continuous monolayer are disrupted and this was in fact accomplished through both MCF-7 spheroid- and 12(S)-HETE-mediated down-regulation of VE-cadherin. This was consistent with the fact that loss of VE-cadherin is associated with a mobile phenotype. VE-cadherin is expressed specifically in endothelial cells and is important for the maintenance and control of endothelial cell contacts. Hence, VE-cadherin is a marker for a differentiated endothelium and an immobile cellular phenotype. Cadherins (E-, P-, N-, M-, and VE-cadherin) are cell adhesion molecules, which organise contacts via Ca^2+^-dependent interactions and bind directly to *β*-catenin, which is required for cohesive function (Vestweber, 2008). Loss of E-cadherin is a key initiating event in EMT (Thiery, 2002). It enables the first step of metastasis – local invasion and dissemination of cancer cells from the primary tumour. ZEB1 is a transcriptional repressor of E-cadherin (Schmalhofer *et al*, 2009) and, therefore, high ZEB1 expression correlates with loss of E-cadherin and an increased migratory and invasive potential and induces EMT (Arumugam *et al*, 2009). Here, we could demonstrate that ZEB1 also regulated 12(S)-HETE-mediated VE-cadherin repression. However, the relation of ZEB1 with VE-cadherin regulation remained unclear. Our results propose that 12(S)-HETE induces an EMT-like phenotype of LECs. This interpretation is problematic, because LECs are of mesenchymal origin yet with an epitheloid phenotype and function.

NF-*κ*B activation was reported to be associated with tumour cell proliferation, survival, angiogenesis, and invasion (Brown *et al*, 2008). Irreversible inhibition of I-*κ*B*α* with Bay11-7082 (Pierce *et al*, 1997) inhibited MCF-7 spheroid-induced CCID formation of LECs in a dose-dependent manner and at low concentration. Since Bay11-7082 caused a decrease of ZEB1 expression and induction of VE-cadherin expression, NF-*κ*B activation is associated with induction of ZEB1 expression (Chua *et al*, 2007). The mode of 12(S)-HETE-induced activation of NF-*κ*B in LECs remains to be established, as we did not observe an increase in E-selectin mRNA levels upon 12(S)-HETE treatment (data not shown). Interestingly, the extracellular addition of S100A4 activates NF-*κ*B through induction of phosphorylation and subsequent degradation of I-*κ*B*α* (Boye *et al*, 2008). We found that 12(S)-HETE-induced S100A4 and Bay11-7082 inhibited S100A4 expression. However, since S100A4 up-regulation occurred after NF-*κ*B-dependent ZEB1 induction, an autocrine activation loop can be excluded. Our study provides biochemical data suggesting that 12(S)-HETE induced a migratory phenotype in LECs (Paulitschke *et al*, 2010) that was already microscopically observed during the formation of large CCIDs in the LEC monolayer underneath MCF-7 spheroids (Madlener *et al*, 2010; Kerjaschki *et al*, 2011). The mechanisms of breast cancer cell intravasation require NF-*κ*B activity that is necessary for LEC motility and the here discovered alterations of LEC structural dynamics allow insights into metastatic mechanisms and the search for anti-metastatic compounds.
